# Investigation of Biogenic Amines and Quality in Jerky, Bacon, and Sausage: Chinese Traditional Meat Product

**DOI:** 10.3390/foods14111842

**Published:** 2025-05-22

**Authors:** Xueying Sun, Xige He, Dan Wang, Min Zhang, Guanhua Hu, Erke Sun, Lina Sun, Ye Jin, Lihua Zhao

**Affiliations:** 1 Department of Food Science, College of Food Science and Engineering, Inner Mongolia Agricultural University, Hohhot 010018, China; sunxueying@imau.edu.cn (X.S.);; 2 Inner Mongolia Key Laboratory for Molecular Regulation of the Cell, Inner Mongolia University, Hohhot 010070, China; 131994998@imu.edu.cn

**Keywords:** jerky, bacon, sausage, biogenic amines, quality

## Abstract

Traditional meat products are renowned for their distinctive flavor and palatability. Nevertheless, the safety of meat products produced by cottage industries remains a matter of concern, especially regarding the content of biogenic amines (BAs) and overall quality. Currently, limited published data exist on these aspects. This study aimed to assess the levels of BAs and key quality index in 41 traditional meat products sourced from China. The analysis revealed that all samples contained measurable levels of total biogenic amines, with concentrations ranging from 11.76 mg/kg to 1632.24 mg/kg. The nitrite content and total volatile basic nitrogen (TVB-N) value of some samples exceeded the standard and normal range. The findings indicate that BAs levels, TBARS values, and nitrite residues of the tested meat products surpassed toxicity thresholds outlined by various regulatory bodies, emphasizing the critical need for enhanced control measures to mitigate biogenic amine content, TBARS, and nitrite residues in meat products.

## 1. Introduction

Chinese traditional meat products encompass both raw and cooked products derived from livestock and poultry and their edible by-products. These products, often incorporating auxiliary ingredients, are crafted through diverse methods including curing, saucing, steaming, boiling, smoking, roasting, baking, drying, frying, and fermentation [1]. This wide array of processing techniques, coupled with China’s expansive geography, has fostered the development of numerous celebrated regional and ethnic meat products, such as sausages, bacon, and jerky. Their distinctive color, aroma, taste, and overall variety contribute significantly to their enduring popularity. However, most traditional meat products are produced in small workshops, where product quality is determined solely by experience and intuition [2]. There is a lack of clear technical indicators and scientific processing theories to guide production, resulting in safety control issues [3]. Food safety is a growing priority, especially within the traditional meat production sector. These products, crafted in natural environments with extended production cycles, create ideal conditions for microbial growth, serving as excellent natural culture media. Due to the high protein and amino acid content of meat products, microorganisms possessing amino acid decarboxylases can cause some amino acids to undergo decarboxylation, resulting in the formation of various biogenic amines [4]. Excessive biogenic amines in food can pose health risks to humans. Therefore, understanding the accumulation of biogenic amines is crucial for ensuring the safety of traditional meat products during processing [5].

Biogenic amines (BAs) are organic compounds that are basic, nitrogen-containing, low-molecular-weight, thermally stable, and non-volatile. These bioactive compounds are found extensively in foods, particularly in high-protein meat products [6]. Biogenic amines are predominantly synthesized through two main pathways. One route involves the amination and transamination reactions of aldehydes or ketones, while the other is mediated by microorganisms, which catalyze the decarboxylation of amino acids [7]. The formation of BAs is contingent upon a confluence of factors. Primarily, the presence of microorganisms possessing amino acid decarboxylase activity is essential. In addition, an adequate supply of free amino acids and oligopeptides is required to act as substrates for enzymatic conversion. Lastly, environmental conditions conducive to the proliferation of these decarboxylase-producing microorganisms are necessary [8,9]. The quantification of BAs in meat products has garnered significant attention due to their established association with adverse health effects, including migraine, hypertension, Parkinson’s disease, and inflammatory responses [10]. Putrescine and cadaverine are considered potential precursors of carcinogenic N-nitroso compounds. Additionally, these biogenic amines serve as indicators of food spoilage [11,12,13].

Individual susceptibility to biogenic amine toxicity exhibits considerable variation, with factors such as genetics, underlying health conditions, the use of drugs that inhibit amino oxidase, and alcohol consumption all influencing individual sensitivity [14]. Currently, there are limited standards for histamine and tyramine across different countries. The Food and Drug Administration (FDA) has set a tolerable histamine threshold for human consumption at 50 mg/kg. EFSA has proposed Acute Reference Dose (ARfD) values for histamine and tyramine, suggesting the following potential ARfD values per adult meal: (1) 50 mg of histamine for healthy individuals, while histamine-intolerant patients have levels below detectable limits, and (2) careful management of tyramine intake. Recommended daily limits are the following: 600 mg for healthy individuals not using Monoamine oxidase (MAO) inhibitors, 50 mg for those taking third-generation MAO inhibitors, and 6 mg for individuals on classic MAO inhibitors [15]. Biogenic amines are known to occur in meat products, including jerky [16], bacon [17], and sausage [18]. Lu et al. conducted a study to measure the biogenic amine content of traditional sausages from various regions of China. They discovered that the levels of tyramine, histamine, cadaverine, and putrescine ranged from 4.96 to 771.52 mg/kg, 0.1 to 101.34 mg/kg, 3.98 to 1435.24 mg/kg, and 0.1 to 449.98 mg/kg, respectively [2]. Biogenic amine content in meat products is largely determined by the quality of raw materials and hygiene during processing and storage [19]. As research progresses, it is anticipated that various countries will establish more detailed limit standards for biogenic amines among various types of food to ensure food safety.

This study addresses a gap in the literature regarding BA levels in meat products sourced from Chinese markets. It investigates the presence and concentration of BAs in representative samples of jerky, bacon, and sausages, evaluating their potential safety. Additionally, the chemical parameters such as TVB-N, nitrite residue, protein content, proteolysis index, non-protein nitrogen, and free amino acids of the traditional jerky, bacon, and sausages were evaluated.

## 2. Materials and Methods

### 2.1. Collection of Samples

Three traditional meat products were collected from retail markets and factories in China, namely jerky (14 samples), bacon (12 samples), and sausage (15 samples), respectively. The sample quantity of each product was 3, and they were from the same batch. The major ingredients of meat products include salt, oil, and spices. As shown in Table 1, the identification codes and ingredient listings for the selected meat product are provided.

### 2.2. Chemicals

Dansyl chloride, 1,7-diaminoheptane, standard BAs, and standard free amino acids (FAAs) were procured from Sigma-Aldrich (a renowned supplier based in St. Louis, MO, USA). The HPLC-grade n-hexane and acetonitrile were supplied by Macklin (Shanghai, China).

### 2.3. Determination of TVB-N, Nitrite Residue, Protein Content

The content of total volatile basic nitrogen (TVB-N) was accurately assayed through the utilization of a Semi-Automatic Kjeldahl nitrogen determinator (KJELTEC-8100, FOSS) following the GB-5009.228-2016 method in China [20]. The chopped sample (5 g) was weighed and combined with 25 mL of trichloroacetic acid. Thoroughly mixed by shaking, the mixture was filtered, and the filtrate was reserved for analysis. To a conical flask containing 10 mL of boric acid solution, 200 μL of mixed indicator solution was added, followed by 10 mL of the pretreated sample filtrate and magnesium oxide. After distillation, the solution was titrated with a standardized titrant until the endpoint, and the titrant volume was recorded.

The residue nitrite (expressed in mg/kg) and protein assessments were carried out in accordance with the GB 5009.33-2010 and GB 5009.5-2016 methods in China [21,22]. A total of 5 g of the sample was combined with 12.5 mL of a 50 g/L saturated borax solution, followed by approximately 150 mL of 70 °C water. It was mixed thoroughly and heated in a boiling water bath for 15 min and cooled in an ice bath to room temperature. The extract was transferred to a 200 mL volumetric flask, and 5 mL of a 106 g/L potassium ferrocyanide solution and 5 mL of a 220 g/L zinc acetate solution were added. The solution was diluted to the mark with water and allowed to stand for 30 min. The upper fat layer was removed and the first 30 mL of filtrate was removed. The absorbance of the remaining filtrate was measured.

### 2.4. Determination of Proteolysis Index (PI)

The non-protein nitrogen (NPN) was quantified according to the method of Hughes et al. [23]. A total of 2.0 g of sample was mixed with 18 mL of distilled water and homogenized for 2 min, followed by centrifugation at 5 °C and 1000 r/min for 15 min. The supernatant was filtered with Whatman No.1 filter paper, repeated once. The volume of the combined filtrate was recorded (V). A total of 15 mL of the filtrate was mixed thoroughly with 15 mL of TCA solution (10% *v*/*v*) and allowed to stand at 25 °C for 0.5 h. After centrifugation and filtration with Whatman No.4 filter paper, 5 mL of the filtrate was collected and the sample nitrogen content N1 was determined using the Kjeldahl method. The non-protein nitrogen content N0 was calculated as N0 = 0.2 × V × N1, where N is the total nitrogen content. The Proteolysis Index (PI) is PI = N0/N.

### 2.5. Free Amino Acid (FAA)

The measurement of free amino acid (FAA) was accomplished with the use of an Automatic Kjeldahl nitrogen determinator (D90324, Beijing Tongrun Yuan Electromechanical Technology Co., Ltd., Beijing, China) in accordance with the GB 5009.124-2016 method in China [24]. A total of 0.05 g of the defatted sample was weighed, 15 mL of hydrochloric acid solution (6 mol/L) was added, and the solution digested at 110 °C for 1 h. After 24 h, it was removed and cooled to room temperature. The initial filtrate was discarded and the other filtrates were collected into a 25 mL volumetric flask. A total of 1 mL of the filtrate was evaporated to dryness under nitrogen at 60 °C. The solution was then acidified with 1 mL of 0.02 mol/L hydrochloric acid, thoroughly mixed, filtered, and the filtrate collected for subsequent instrumental analysis.

### 2.6. Biogenic Amines (BAs)

The determination of BAs was conducted through extraction and derivatization procedures, which were adapted from the methods described by Sun et al. [25]. Specifically, 5 g of sample was first extracted with 5% trichloroacetic acid. Subsequently, hexane was employed to remove the fat component. For further purification, a 1:1 mixture of chloroform and n-butanol was utilized. Following purification, the samples were derivatized with dansyl chloride. After derivatization, each sample was filtered through a 0.22 μm filter, and a 10 µL aliquot of the filtrate was injected into a chromatographic column (ZORBAX SB-C18, 250 mm × 4.6 mm, 5 µm particle size, Agilent) of a high-performance liquid chromatograph (1260; Agilent, Santa Clara, CA, USA) with a UV detector (1260VWD). The separated biogenic amines were identified based on the retention times of the known standard, 1,7-diaminoheptane. Quantification of BAs in the samples was achieved by interpolation using the calculated regression lines based on an internal standard method. Results of regression equations are presented in Table S1. Finally, the content of BAs was expressed as milligrams per kilogram (mg/kg) on a fresh matter basis.

### 2.7. Statistical Analysis

All experimental data are presented as mean ± standard deviation. The data were analyzed using SPSS 26.0 (IBM, Chicago, IL, USA). One-way analysis of variance (ANOVA) was conducted to evaluate the statistical significance of the data. A post hoc comparison LSD test was used to determine significant differences. The graphical representations shown in this article were generated with Origin 2019.

## 3. Results

### 3.1. Protein Content and Proteolytic Index (PI) of Traditional Meat Products

**Figure 1:**
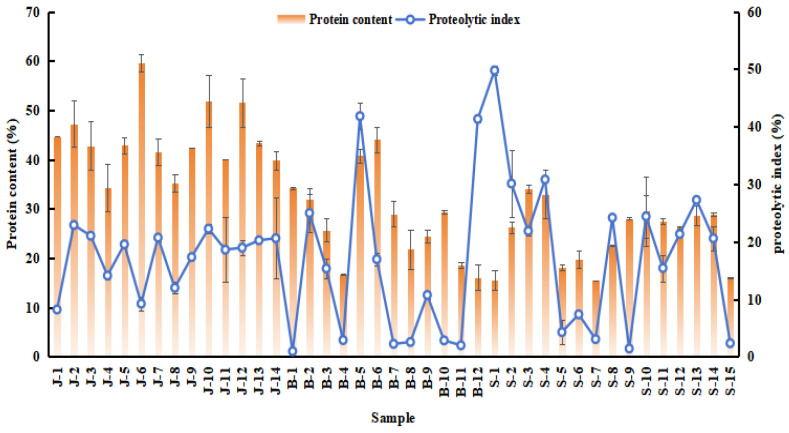
Quantity of different protein content (%) and Proteolysis Index in the 41 meat products from the traditional cottage industry. J: jerky, B: bacon, S: sausage.

The protein content of meat products is a crucial parameter for assessing their nutritional value, and PI represents the percentage of non-protein nitrogen to total nitrogen in traditional meat products, indicating the protein hydrolysis of these products. The results are shown in Figure 1. The crude protein content of jerky from various regions was generally higher, with almost all samples exceeding 35% (J1–J14). Bacon displayed a wider range (16.12–44.06%), with Jinhua bacon (B6) showing a significantly elevated protein level. Chengdu sausage (S3, 33.99%) also presented a higher protein content compared to other sausage samples. Overall, the protein content of jerky was generally greater than that of other samples, and the variability in protein content among bacon and sausage samples was not significant. The selection of raw meat and the processing methods are closely linked to the protein content of meat products. More specifically, the protein content of beef is higher than that of pork, leading to significant variations in the nutritional value of jerky, bacon, and sausage across different regions. The protein index (PI) varied significantly across samples: jerky (9.20–22.89%), bacon (0.93–41.82%), and sausage (1.39–49.77%). This indicates that there is no strong correlation between the protein content of the samples and the protein index. The degree of protein hydrolysis in jerky was lower than in the other two types of meat products, although the free amino acid composition ratios were similar. This may be attributed to the lower water activity (a_w_) in jerky and bacon samples, which inhibited the activity of protein-hydrolyzing enzymes. In contrast, the higher moisture content of sausage promoted enzyme activity and subsequent protein catabolism, resulting in elevated non-crude protein nitrogen content [26].

### 3.2. Analysis of Free Amino Acid Content in Traditional Meat Products from Different Regions

**Figure 2:**
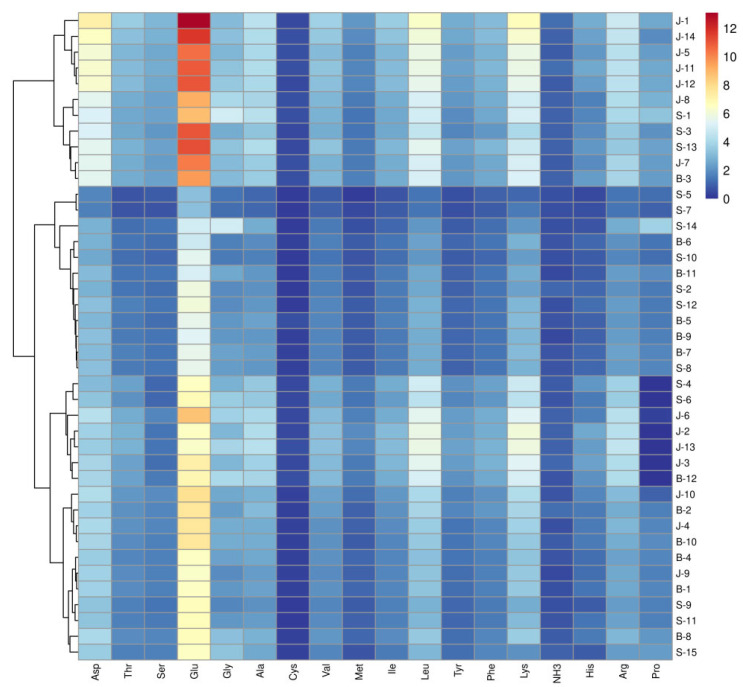
Heatmap of different free amino acid in the 41 meat products from the traditional cottage industry. J: jerky, B: bacon, S: sausage.

Free amino acids, derived from muscle protein degradation, serve as precursors for flavor compound formation through pathways like the Maillard reaction and Strecker degradation. These compounds ultimately define the final flavor profile [27]. The amino acid content of meat products from various regions is illustrated in Figure 2. A total of 17 free amino acids were recognized in traditional meat products, among which 7 were essential amino acids and 10 were non-essential amino acids. Among the three types of traditional meat products, jerky exhibited the highest concentration of essential amino acids, followed by sausages, with a significant difference in the content of samples from different regions within the same category (*p* < 0.05). The average proportion of essential amino acids in jerky exceeded 40%, and there was no significant difference in the content of essential amino acids in jerky from different regions (*p* > 0.05). The average proportion of bacon and sausage exceeded 37%. The proportion of free amino acids in the three types of meat products was similar, with the primary components being fresh amino acids (Glu, Asp), bitter amino acids (Lys, Leu, Arg), and sweet amino acids (Ala, Gly).

### 3.3. Analysis of Sodium Nitrite Residue Amount and TVB-N in Traditional Meat Products from Different Regions

**Figure 3:**
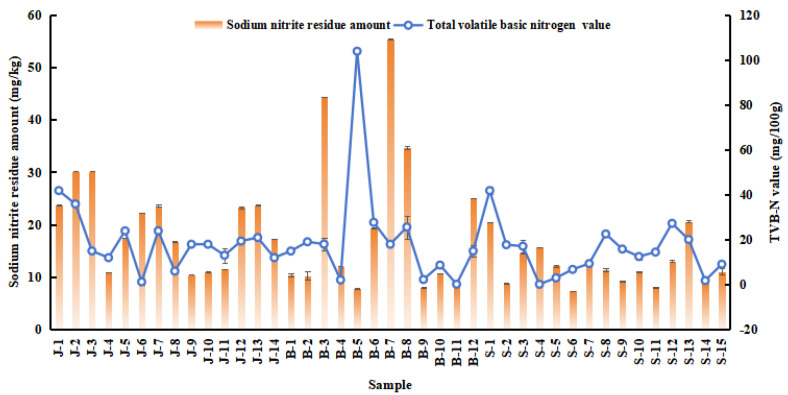
Quantity of different sodium nitrite residue amount and TVB-N value in the 41 meat products from the traditional cottage industry. J: jerky, B: bacon, S: sausage.

Nitrite, a key additive in meat processing, contributes to color and flavor enhancement, microbial inhibition, oxidation prevention, and corrosion protection. Analysis of three meat product types (Figure 3) revealed residual sodium nitrite levels ranging from 7.32 to 55.40 mg/kg. It was found that five samples (jerky from J2 and J3, bacon from B3, B7, and B8) exceeded the standard limit, while most traditional meat products contained sodium nitrite residues below the national standard method (GB 5009.33–2016) in China [28]. The residual nitrite levels in jerky were higher than those in bacon and sausage. Sausage samples from various regions exhibited very low residual sodium nitrite levels, whereas jerky samples had elevated nitrite levels due to the dried meat production process. The necessity to maintain color and inhibit bacteria in meat products results in the addition of more nitrite during production. Consequently, the safety of traditional meat products must be ensured in future meat processing practices.

TVB-N refers to the collective group of basic metabolites, like ammonia and biogenic amines (BAs), which are synthesized as a result of the metabolic processes carried out by microorganisms that break down nitrogen-containing compounds like proteins and amino acids [29]. TVB-N is frequently used as a biomarker for protein and amine degradation in meat products to assess meat freshness [30]. A higher TVB-N value indicates more extensive destruction of amino acids, significantly affecting the nutritional value and flavor quality of the product. Residual nitrite levels in all samples ranged from 0.12 to 103.73 mg/100 g (Figure 3). With the exception of Inner Mongolia bacon, which had TVB-N values exceeding national standards, all other samples fell within the normal range.

### 3.4. Analysis of BAs in Traditional Meat Products from Different Regions

**Figure 4:**
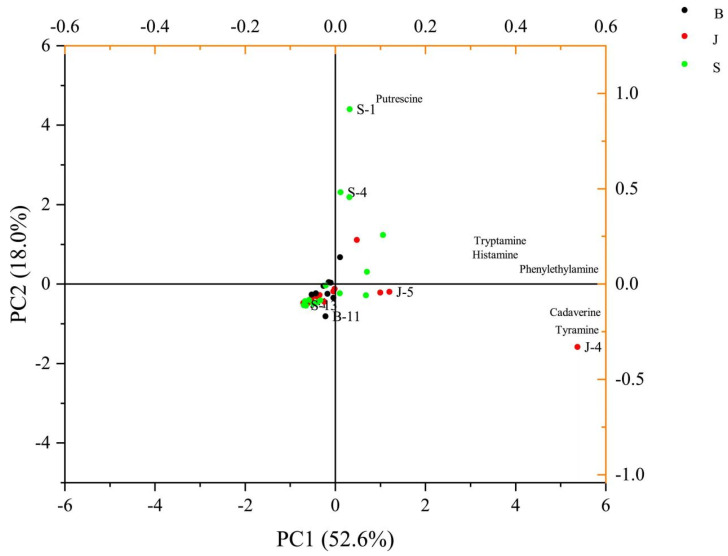
The PCA of the BA levels of traditional meat product. B: bacon; J: jerky; S: sausage.

Beef, bacon, and sausage produced in the traditional household handicraft industry contain complex spices, which are fermented by various microorganisms, making them more susceptible to biogenic amine (BA). Among fermented foods, histamine, tyramine, cadaverine, 2-phenylethylamine, and putrescine are the most commonly identified and principal biogenic amines [31]. As biogenic amines (BAs) are significant toxins in fermented foods, the biogenic amine content of 41 kinds of handmade products from different regions across the country was evaluated. Moreover, it was explored how they influence the quality and safety of these products. As presented in Table 2, the total content of biogenic amines (BAs) in all traditional meat samples spanned from 11.76 mg/kg to 1632.24 mg/kg, showing notable variations among different samples. In a similar study, Lu et al. examined biogenic amines in 42 traditional Chinese sausage samples sourced from various origins. Their results identified cadaverine as the predominant amine, followed by tyramine and putrescine. Some samples exhibited a total biogenic amine content exceeding 1000 mg/kg [2]. Principal Component Analysis (PCA), detailed in Figure 4, assessed BA production across samples. PC1 and PC2 accounted for 52.6% and 18.0% of the variance, respectively, totaling 70.6%. This suggests limited variation in BA profiles overall. The tyramine and cadaverine levels notably affected products J-4 and J-5, while the histamine, cadaverine, 2-phenylethylamine, and putrescine levels influenced products S-1 and S-4. However, a few meat samples exhibited no variation in BA quantities (Figure 4).

The highest concentration of BAs was observed in J4 (1632.24 ± 28.40 mg/kg), B5 (255.92 ± 15.09 mg/kg), and S6 (490.05 ± 16.76 mg/kg) across three types of meat products. Based on the total biogenic amine (BA) content, the samples were categorized into three distinct groups. Group I comprised samples with BA levels below 75 mg/kg (indicating non—detection); Group II included samples with BA concentrations ranging from 75 to 100 mg/kg; and Group III consisted of samples with BA levels exceeding 100 mg/kg. The largest proportion of meat samples, accounting for 58.54%, belonged to Group III. While there are currently no rigid regulatory standards regarding the total content of biogenic amines (BAs) in fermented foods, numerous studies indicate that 1000 mg/kg is considered the upper threshold for acceptable levels [7]. The BAs in J4 exceed the recommended limit (<1000 mg/kg), and the variation in BA content among the different meat products can be attributed to several factors; however, the wide range in total biogenic amine content in meat products indicates that there are significant differences in the total biogenic amines in meat products produced under different processes and processing environments. Research has found that factors such as the quality and type of raw materials, storage duration and temperature, as well as different microbial communities all influence the accumulation of biogenic amines [32,33]. Researchers indicate that *Pseudomonas* and *Acinetobacter* are dominant bacterial genera in traditional meat products [16]. *Pseudomonas* is a key spoilage organism; exceeding a threshold population level of *Pseudomonas* can readily induce product spoilage [34]. This is because *Pseudomonas* species are psychrophilic aerobic bacteria with strong metabolic capabilities and can secrete siderophores, which inhibit the growth of other microorganisms, allowing them to gradually become the dominant species [35]. Mansur found that *Pseudomonas* is the main bacterial community in beef, with volatile organic compounds such as ethyl acetate, diacetyl, and 2-ethylhexanol being associated with meat off-flavors likely produced by this bacterium [36]. *Acinetobacter* uses amino acids as substrates, has a low spoilage ability, and does not produce off-odors during amino acid degradation. However, once it becomes the dominant spoilage bacterium, it can enhance the spoilage ability of *Pseudomonas*. The workshop environment and the equipment used during processing are the main factors causing contamination of soy-sauce-marinated meat products with these bacteria during production [37]. These bacteria are considered good producers of BAs due to the presence of decarboxylases [32]. Decarboxylase activity varies significantly among different strains, which may be the main reason for the differences in BA content in various meat products [38]. In addition to bacteria, some studies have also reported that yeasts can produce BAs [39].

Tyramine, a biogenic amine present in food, raises concerns due to its involvement in cheese reactions and its potential to induce syndromes such as poisoning [40]. While regulatory standards for tyramine levels remain undefined, available data suggest a potential toxicity threshold ranging from 100 to 800 mg/kg [41]. In this study, tyramine was detected in the tested meat samples at levels ranging from 0 to 576.47 mg/kg (Table 2). A number of samples surpassed the toxicity thresholds recommended by regulatory authorities. Based on the tyramine levels, the gochujang samples were meticulously stratified into three distinct categories: Group I, encompassing samples with tyramine concentrations ranging from non-detectable levels up to 5 mg/kg; Group II, characterized by tyramine content between 5 and 20 mg/kg; and Group III, consisting of samples with tyramine levels exceeding 20 mg/kg. The majority of meat samples were assigned to Group I, representing 39.02% of the total, followed by Group II (29.27%) and Group III (31.71%). Notably, the tyramine concentrations within these samples remained well beneath the established toxicity thresholds, which typically span from 100 to 800 mg/kg. These findings are in remarkable congruence with the data reported by Lu et al. (2010), who documented tyramine levels ranging from 1.0 to 18.31 mg/kg in 42 sausage samples [2].

Histamine has garnered significant attention among various biogenic amines, primarily due to its potent toxic effects, such as scombroid poisoning [42]. The World Health Organization (WHO) has meticulously established a stringent maximum permissible limit of 200 mg/kg for histamine in food products. Analogously, regulatory bodies in Korea and China have put forth a recommended range of 200–400 mg/kg as the upper threshold for histamine content in food items. In the current study, a comprehensive analysis of 41 meat products was conducted. The findings revealed that histamine levels among these samples exhibited significant variability, spanning from non-detectable concentrations to a peak of 135.64 mg/kg, as detailed in Table 2. The peak concentration of histamine was observed in sample J13, whereas the lowest levels were detected in samples J1, J11, and S9–S11. In accordance with the histamine content, the specimens were systematically classified into three distinct categories: Group I, encompassing samples with histamine concentrations ranging from non-detectable values up to 5 mg/kg; Group II, characterized by histamine levels between 5 and 10 mg/kg; and Group III, comprising samples with histamine concentrations exceeding 10 mg/kg. A majority of the meat samples, specifically 51.22%, were assigned to Group I, followed by 46.34% in Group III and a mere 2.44% in Group II. Significantly, the histamine levels within all samples remained well below the toxicity thresholds recommended by multiple regulatory bodies, strongly suggesting that, based on histamine content, the 41 meat products were deemed safe for human consumption. These results are in excellent concordance with the findings of previous research conducted by Gong et al., which reported comparable histamine levels across a diverse array of fermented samples [3].

Putrescine, cadaverine, tryptamine, and phenylethylamine are common biogenic amines (BAs) found in food. While no legal limits currently exist for putrescine and cadaverine due to their comparatively weaker toxicity, their presence can exacerbate the effects of histamine and tyramine by inhibiting their breakdown [43]. Among the 41 meat product samples evaluated in this article, putrescine, cadaverine, tryptamine, and phenylethylamine were detected within the ranges of 0–258.96 mg/kg, 0–446.79 mg/kg, 1.5148–203.87 mg/kg, and 0–397.09 mg/kg, respectively (Table 2).

A considerable amount of BAs was found in jerky (J4, J5), bacon (b3, b5), and sausage (S1, S6). These samples contained high levels of free amino acids, which may have influenced the formation of BAs in products since it serves as a substrate for the synthesis of various types of BAs [44]. The biogenic amine content in Samples J1, J3, J11, B2, B12, S2, and S7 was relatively low, possibly due to the presence of chili, pepper, and spices, which may explain the lower levels of BAs in meat products [31,45]. Two factors together determine the content of biogenic amines by inhibiting microbial growth and reducing amino acid decarboxylase activity in meat products. In addition, researchers have found that many microorganisms have the ability to degrade biogenic amines, which can be applied to control the content of biogenic amines in food. The biogenic amine degradation ability of microorganisms is attributed to amine-degrading enzymes, mainly including amine oxidases and multicopper oxidases [46]. Zhang found that starters producing amine oxidase can reduce the formation of biogenic amines during sausage processing [47]. Therefore, to control the biogenic amine content in meat products, in addition to managing the production environment, temperature, and conditions, it is also possible to add spices or starters containing amine oxidase during the processing of the products to improve product quality.

## 4. Conclusions

This study detected biogenic amines and chemical parameters in all 41 traditional meat samples from China. Analyzed sausages, bacon, and cured meats exhibited elevated biogenic amine levels, particularly tyramine and putrescine. BA levels, TBARS values, and nitrite residues in some meat products exceeded the toxicity limits recommended by various regulatory bodies. Therefore, it can be concluded that since the meat products analyzed in this study may have certain effects on human health, controlling biogenic amine content in these products is crucial.

## Figures and Tables

**Table 1:** Details of Traditional Jerky, Bacon and Sausages.

Product	Sample	Location of the Company (China)	Latitude and Longitude	Ingredients
Jerky	J-1	Alxa Left Banner	E105.67, N38.83	vegetable oil, edible salt, spices
	J-2	Alxa Right Banner	E101.67, N39.22	vegetable oil, edible salt, spices
	J-3	Ejin Banner	E101.06, N41.95	vegetable oil, edible salt, spices
	J-4	Ordos	E116.41, N39.91	vegetable oil, edible salt
	J-5	Dongsheng District	E109.96, N39.82	vegetable oil, edible salt
	J-6	Dalad Banner	E110.03, N40.41	vegetable oil, edible salt
	J-7	Tsining District	E113.17, N41	vegetable oil, edible salt, spices
	J-8	Urad Front Banner	E113.21, N40.78	vegetable oil, edible salt, spices
	J-9	Xilingol League	E116.05, N43.93	vegetable oil, edible salt, spices
	J-10	Zhenglan Banner	E115.99, N42.24	vegetable oil, edible salt, spices
	J-11	Bordered Yellow Banner	E113.85, N42.23	vegetable oil, edible salt, spices
	J-12	Ulan Hot	E122.09, N46.07	edible salt, aginomoto, spices
	J-13	Hinggan League	E122.04, N46.08	vegetable oil, edible salt, spices
	J-14	Ili	E81.32, N43.92	salt, vegetable oil, monosodium glutamate, soy sauce, additives
Bacon	B-1	Hubei	E109.48, N30.3	Chinese prickly ash, edible salt, cypress twig, cinnamon, orange peel
	B-2	Chengdu	E104.07, N30.57	salt, wine, spices, food additives
	B-3	Guizhou	E106.63, N26.65	edible salt, spices
	B-4	Hunan	E112.94, N28.23	edible salt, spices
	B-5	Inner Mongolia	E111.77, N40.82	edible salt
	B-6	Jinhua	E119.65, N29.08	edible salt, additives
	B-7	Xuanwei	E101.76, N25.19	sugar, salt, wine, spices, additives
	B-8	Longxi	E104.63, N35	clove, amomum villosum, salt, zanthoxylum bungeanum, ginger peel, daxiang, caoshao, fennel, wine, spices
	B-9	Zhejiang	E120.58, N30.05	sugar, salt, wine, monosodium glutamate, additives
	B-10	Guangdong	E110.36, N21.27	sugar, salt, wine, soy sauce, food additives
	B-11	Anhui	E117.23, N31.82	edible salt
	B-12	Yunnan	E102.83, N24.88	edible salt, spices
Sausage	S-1	Xinjiang	E87.63, N43.79	edible salt
	S-2	Hubei	E109.48, N30.3	salt, wine, sugar, monosodium glutamate, chili, pepper
	S-3	Chengdu	E104.07, N30.57	salt, sugar, chili, pepper, spices
	S-4	Guizhou	E106.63, N26.65	sugar, salt, pepper, monosodium glutamate
	S-5	Guangdong	E110.36, N21.27	soybean protein powder, sugar, salt, wine, gluten powder
	S-6	Anhui	E117.33, N31.73	sugar, salt, Baijiu, additives
	S-7	Harbin	E126.54, N45.8	salt, sugar, monosodium glutamate, garlic, spices
	S-8	Jiangxi	E114.97, N27.09	salt, sesame oil, spices
	S-9	Laiwu	E117.68, N36.21	soy sauce, soy protein powder, salt, spices
	S-10	Harbin	E126.54, N45.8	sugar, wine, salt, star anise, pepper
	S-11	YI Li	E118.36, N35.1	salt, sugar, soy sauce, monosodium glutamate, spices, chicken essence
	S-12	Ulan River	E121.95, N46.08	vegetable oil, edible salt, spices
	S-13	Xingan league	E122.09, N46.07	vegetable oil, edible salt, spices
	S-14	Zhejiang	E120.58, N30.05	edible salt, additives
	S-15	YiYang	E112.36, N28.55	edible salt

**Table 2:** Quantity of different biogenic amines (mg/kg) in the 41 traditional meat products (±0.01 means standard deviation; when the measured values of all three replicates were below the LOD, the results were reported as <LOD).

Product	Sample	Tryptamine	Phenylethylamine	Putrescine	Cadaverine	Histamine	Tyramine	Total Biogenic Amine
Jerky	J1	9.77 ± 0.01	0.47 ± 0.01	1.08 ± 0.00	<LOD	ND	0.44 ± 0.00	11.76 ± 0.01
	J2	41.38 ± 1.80	78.45 ± 3.51	8.48 ± 0.42	31.70 ± 1.42	10.19 ± 1.72	10.40 ± 0.42	180.62 ± 9.27
	J3	10.68 ± 0.00	0.21 ± 0.00	ND	11.31 ± 0.43	8.04 ± 0.34	0.49 ± 0.00	30.73 ± 0.77
	J4	111.65 ± 9.35	397.09 ± 3.30	12.54 ± 0.55	446.79 ± 6.90	87.7 ± 15.71	576.47 ± 3.26	1632.24 ± 27.00
	J5	71.55 ± 2.04	137.23 ± 2.95	22.77 ± 0.56	141.62 ± 2.58	30.61 ± 13.25	113.51 ± 3.66	517.29 ± 11.75
	J6	59.01 ± 3.15	118.06 ± 1.74	19.53 ± 0.38	127.86 ± 4.06	38.7 ± 0.84	95.78 ± 1.78	458.94 ± 5.90
	J7	22.31 ± 0.60	ND	7.29 ± 0.18	8.55 ± 0.21	4.93 ± 0.14	20.47 ± 0.52	63.56 ± 1.23
	J8	19.23 ± 0.01	14.75 ± 0.10	1.92 ± 0.01	5.62 ± 0.04	37.33 ± 0.10	12.60 ± 0.07	91.45 ± 0.22
	J9	12.72 ± 0.02	4.60 ± 0.02	1.64 ± 0.01	3.14 ± 0.03	3.36 ± 0.010	6.53 ± 0.01	31.98 ± 0.04
	J10	34.00 ± 0.73	7.87 ± 0.15	4.72 ± 0.02	11.26 ± 0.13	11.07 ± 0.06	15.82 ± 0.01	84.74 ± 0.78
	J11	2.60 ± 0.02	13.93 ± 0.30	3.49 ± 0.01	3.14 ± 0.03	ND	1.17 ± 0.00	24.33 ± 0.28
	J12	28.06 ± 2.44	22.06 ± 1.86	3.28 ± 0.27	19.25 ± 1.69	11.75 ± 0.95	48.77 ± 4.50	133.17 ± 11.68
	J13	39.99 ± 1.52	29.40 ± 1.38	55.03 ± 2.89	19.59 ± 0.94	135.64 ± 6.50	26.45 ± 1.97	306.10 ± 15.07
	J14	45.9 ± 0.21	118.2 ± 0.55	6.57 ± 0.05	7.75 ± 0.03	1.39 ± 0.00	7.30 ± 0.01	187.11 ± 0.79
Bacon	B1	9.61 ± 0.39	12.49 ± 0.58	18.53 ± 0.89	18.7 ± 0.99	70.99 ± 3.61	28.95 ± 1.38	159.26 ± 7.75
	B2	17.37 ± 0.03	2.46 ± 0.01	<LOD	1.39 ± 0.02	1.90 ± 0.02	1.43 ± 0.01	24.55 ± 0.04
	B3	19.62 ± 1.77	117.59 ± 10.74	22.26 ± 2.06	1.43 ± 0.24	1.49 ± 0.16	5.74 ± 0.58	168.12 ± 15.52
	B4	29.29 ± 0.03	7.80 ± 0.07	14.48 ± 0.00	61.55 ± 0.01	48.54 ± 0.03	56.79 ± 0.03	218.45 ± 0.17
	B5	66.09 ± 0.45	49.39 ± 0.58	55.25 ± 2.40	14.47 ± 0.23	16.27 ± 0.63	54.45 ± 10.02	255.92 ± 12.32
	B6	12.04 ± 0.08	3.49 ± 0.09	0.65 ± 0.01	1.47 ± 0.02	0.88 ± 0.02	2.98 ± 0.04	21.51 ± 0.16
	B7	63.89 ± 0.59	3.14 ± 0.06	0.61 ± 0.03	3.40 ± 0.03	1.85 ± 0.51	2.07 ± 0.32	74.96 ± 0.54
	B8	80.49 ± 0.77	8.62 ± 0.16	0.48 ± 0.01	74.81 ± 0.66	4.94 ± 0.12	2.77 ± 0.05	172.11 ± 1.70
	B9	5.76 ± 0.05	18.00 ± 0.01	15.81 ± 0.02	33.54 ± 0.05	37.78 ± 0.11	43.25 ± 0.16	154.14 ± 0.27
	B10	18.59 ± 0.19	19.87 ± 0.54	13.18 ± 0.47	6.14 ± 0.21	46.67 ± 2.44	15.21 ± 0.31	119.65 ± 3.72
	B11	8.15 ± 0.03	5.39 ± 0.71	2.07 ± 0.01	121.09 ± 0.81	0.15 ± 0.00	3.84 ± 0.00	140.69 ± 0.14
	B12	15.75 ± 0.61	27.61 ± 0.56	10.28 ± 0.05	2.26 ± 0.61	2.65 ± 0.09	5.24 ± 0.87	63.79 ± 1.69
Sausage	S1	63.68 ± 1.04	80.84 ± 1.42	258.96 ± 4.18	3.91 ± 0.06	35.51 ± 0.65	7.18 ± 0.24	450.08 ± 7.50
	S2	5.25 ± 0.59	6.50 ± 0.12	<LOD	10.50 ± 0.02	0.57 ± 0.02	3.89 ± 0.04	26.71 ± 0.75
	S3	3.55 ± 0.46	64.11 ± 0.39	1.04 ± 0.02	12.27 ± 0.07	4.65 ± 0.14	2.09 ± 0.09	88.05 ± 0.53
	S4	18.90 ± 0.05	127.79 ± 0.63	153.61 ± 0.21	17.88 ± 0.31	11.45 ± 0.47	1.46 ± 0.05	331.09 ± 1.70
	S5	29.29 ± 0.03	7.80 ± 0.07	14.48 ± 0.00	61.55 ± 0.01	48.54 ± 0.03	56.79 ± 0.03	218.45 ± 0.17
	S6	203.87 ± 0.69	134.68 ± 17.81	51.79 ± 0.26	ND	3.63 ± 0.06	96.08 ± 0.02	490.05 ± 16.77
	S7	1.51 ± 0.00	10.84 ± 0.02	4.64 ± 0.01	<LOD	3.50 ± 0.01	3.70 ± 0.01	24.42 ± 0.03
	S8	127.62 ± 3.99	25.80 ± 0.13	1.58 ± 0.07	69.80 ± 0.09	92.73 ± 0.28	9.09 ± 0.24	326.62 ± 3.73
	S9	7.94 ± 0.92	36.82 ± 3.48	1.66 ± 0.43	20.08 ± 0.73	ND	1.86 ± 0.25	68.36 ± 5.57
	S10	11.95 ± 0.04	73.71 ± 0.30	2.63 ± 0.02	11.53 ± 0.07	ND	9.27 ± 0.32	109.09 ± 0.71
	S11	105.74 ± 0.05	9.78 ± 0.12	1.69 ± 0.02	9.80 ± 0.12	ND	ND	127.01 ± 0.20
	S12	10.37 ± 0.07	88.21 ± 19.67	157.81 ± 35.16	84.51 ± 18.74	32.34 ± 7.55	8.36 ± 2.02	381.60 ± 83.22
	S13	1.94 ± 0.02	4.31 ± 0.11	0.58 ± 0.02	3.90 ± 0.22	0.48 ± 0.00	3.67 ± 0.01	14.88 ± 0.30
	S14	43.94 ± 1.15	45.08 ± 4.65	ND	62.70 ± 0.08	92.64 ± 0.35	101.72 ± 7.75	346.08 ± 13.13
	S15	14.60 ± 2.68	15.40 ± 5.03	3.20 ± 0.11	3.33 ± 1.7	3.70 ± 1.37	2.78 ± 0.4	43.00 ± 11.29

## Data Availability

The original contributions presented in this study are included in the article/Supplementary Materials. Further inquiries can be directed to the corresponding author.

## Supplementary Materials

The following supporting information can be downloaded at: https://www.mdpi.com/article/10.3390/foods14111842/s1, Table S1: Result of regression equations.

## Abbreviations

The abbreviations listed below are used for brevity and clarity in this manuscript:

MAO	Monoamine oxidase
BAs	Biogenic amines
FAA	Free amino acid
NPN	Non-protein nitrogen
PI	Proteolysis index
TVB-N	Total volatile basic nitrogen
