# Supplementary material for: Investigation of Biogenic Amines and Quality in Jerky, Bacon, and Sausage: Chinese Traditional Meat Product
Source: Foods. 2025 May 22;14(11):1842. doi: 10.3390/foods14111842 (PMC12154260; doi:10.3390/foods14111842)
Supplement: Supplementary file 1 [file foods-14-01842-s001.zip › foods-3609376-supplementary.pdf]

Table S1. Result of regression equations. 1

Biogenic Amines	Regression Equation	Correlation coefficient R^2	Linear Range (mg/L)	Peak Time	LOD (mg/L)	LOQ (mg/L)
Putrescine	y = 0.0431x +0.0364	0.9994	1 to 50	17.431	0.23	0.77
Cadaverine	y = 0.0264x +0.0028	0.9999	1 to 50	19.021	0.25	0.83
Tyramine	y = 0.0211x +0.0031	0.9994	1 to 50	27.438	0.28	0.98
Histamine	y = 0.0173x +0.0151	0.9993	1 to 50	19.955	0.15	0.45
Tryptamine	y = 0.0092x +0.0009	0.9997	1 to 50	13.146	0.16	0.56
Phenylethylamine	y =0.0082x - 0.0002	0.9995	1 to 50	15.811	0.12	0.39

2
